# Ergosterol purified from medicinal mushroom *Amauroderma rude* inhibits cancer growth *in vitro* and *in vivo* by up-regulating multiple tumor suppressors

**DOI:** 10.18632/oncotarget.4026

**Published:** 2015-05-27

**Authors:** Xiangmin Li, Qingping Wu, Yizhen Xie, Yinrun Ding, William W. Du, Mouna Sdiri, Burton B. Yang

**Affiliations:** ^1^ School of Bioscience and Bioengineering, South China University of Technology, Guangzhou 510006, PR China; ^2^ State Key Laboratory of Applied Microbiology Southern China (The Ministry-Province Joint Development), Guangdong Institute of Microbiology, Guangzhou, 510070, PR China; ^3^ Sunnybrook Research Institute, Sunnybrook Health Sciences Centre, Toronto, M4N3M5, Canada; ^4^ Department of Laboratory Medicine and Pathobiology, University of Toronto, Toronto, M4N3M5, Canada

**Keywords:** herbal medicine, medicinal mushroom, Foxo3a, Bim, Fas

## Abstract

We have previously screened thirteen medicinal mushrooms for their potential anti-cancer activities in eleven different cell lines and found that the extract of *Amauroderma rude* exerted the highest capacity in inducing cancer cell death. The current study aimed to purify molecules mediating the anti-cancer cell activity. The extract of *Amauroderma rude* was subject to fractionation, silica gel chromatography, and HPLC. We purified a compound and identified it as ergosterol by EI-MS and NMR, which was expressed at the highest level in *Amauroderma rude* compared with other medicinal mushrooms tested. We found that ergosterol induced cancer cell death, which was time and concentration dependent. In the *in vivo* experiment, normal mice were injected with murine cancer cell line B16 that is very aggressive and caused mouse death severely. We found that treatment with ergosterol prolonged mouse survival. We found that ergosterol-mediated suppression of breast cancer cell viability occurred through apoptosis and that ergosterol up-regulated expression of the tumor suppressor Foxo3. In addition, the Foxo3 down-stream signaling molecules Fas, FasL, BimL, and BimS were up-regulated leading to apoptosis in human breast cancer cells MDA-MB-231. Our results suggest that ergosterol is the main anti-cancer ingredient in *Amauroderma rude*, which activated the apoptotic signal pathway. Ergosterol may serve as a potential lead for cancer therapy.

## INTRODUCTION

Drug discovery and development for therapeutics of various diseases including cancer are still reliant on natural ingredients. In cancer treatment, many drugs have natural origins or are obtained directly from natural sources [1–3]. Microorganisms have been continuous sources for drug development as some bioactive molecules can be easily obtained from these species. Medicinal mushrooms, used in oriental traditional therapies, contain many functional compounds to inhibit tumor growth [4–7].

*Ganodermataceae* is a well-known family of medicinal mushrooms, which contains 11 genus including Ganoderma and Amauroderma. *Ganoderma lucidum*, a traditional Chinese medicinal fungus used for over 2000 years, is the most known medicinal mushroom in Ganoderma and is regarded as folk medicine used for prevention and treatment of various human diseases, including cancer [8–13]. The genus Amauroderma contains approximately 30 species and most of the species are widespread in tropical areas [14]. *Amauroderma rude* (Berk.) Torrend (called ‘Xuezhi’ in China) and some species in this genus have been newly recognized as medicinal fungus [15–17]. Techniques have been developed to cultivate *Amauroderma rude* making it possible to obtain large quantity of *Amauroderma rude* [16].

Over the past decades, it has been demonstrated that many fungus compounds exert anti-cancer activity by boosting immunity or directly inducing cancer cell death [18–20]. The main bioactive compounds are polysaccharides, terpenoids, and sterols [21–23]. Polysaccharides, which have been isolated from *Ganoderma lucidum, Cordyceps sinensis, Lentinula edodes, Coriolus versicolor, Grifola frondosa, Schizophyllum commune*, possess anti-cancer activity mainly through activating the immune system and facilitating the immune cells to attack cancer cells [24–27]. Lentinan from *Lentinula edodes*, Polysaccharide Krestin (PSK) and Polysaccharide Peptide (PSP) from *Coriolus versicolor* have been used in clinics for several decades [22, 28]. We have previously reported that the water extract of *Amauroderma rude* inhibited cancer cell survival and induced cell apoptosis [16]. Since polysaccharides may be the major components in the water extract that possess anti-cancer activity, small molecules such as terpenoids and sterols may stay in the lipid fraction. In this study, we aim to identify small molecules in the lipid fraction of *Amauroderma rude* with anti-cancer activity.

## RESULTS

### Ethanol extract and chloroform fraction of *Amauroderma rude* induced cancer cell death

**Figure 1:**
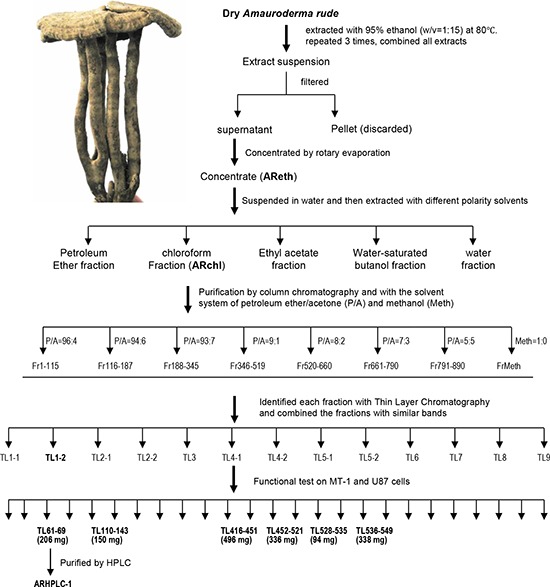
Purification of anti-cancer ingredient and molecule from *Amauroderma rude* The dry fruit bodies of *Amauroderma rude* were extracted using 95% ethanol. The extract was subject to chromatography and HPLC purification as indicated. Human breast cancer cell cultures were used to screen the anti-cancer ingredients in each step until single pick was obtained from HPLC. AReth, ethanol extract of *Amauroderma rude*; ARchl, chloroform fraction of *Amauroderma rude*; P/A, ratios of petroleum ether to ether in the elution buffer; Meth, pure methanol used to elute the column; Fr, fraction number; TL, fractions obtained from the Thin layer Chromatography; inset, photo of a dry *Amauroderma rude*.

We have previously reported that the water extract of *Amauroderma rude* inhibited growth of cancer cells [16]. In this study, we aimed to identify the anti-cancer compound by a number of purification approaches. The anti-cancer activity of each component was monitored by incubating with cancer cell cultures. Using this approach, we found that the ethanol extract (AReth) was very potent in inducing cancer cell death. The ethanol extract was then fractionated by petroleum ether, chloroform, ethyl acetate, water-saturated butanol, and water alone. After evaporation of the collection, petroleum ether fraction (PEF), chloroform fraction (ARchl), ethyl acetate fraction (EAF), water-saturated butanol fraction (BF), and water fraction (WF) were obtained (Fig. 1). We found that the chloroform fraction (ARchl) displayed the highest activity in inducing cancer cell death. A total of 20 grams was obtained.

**Figure 2:**
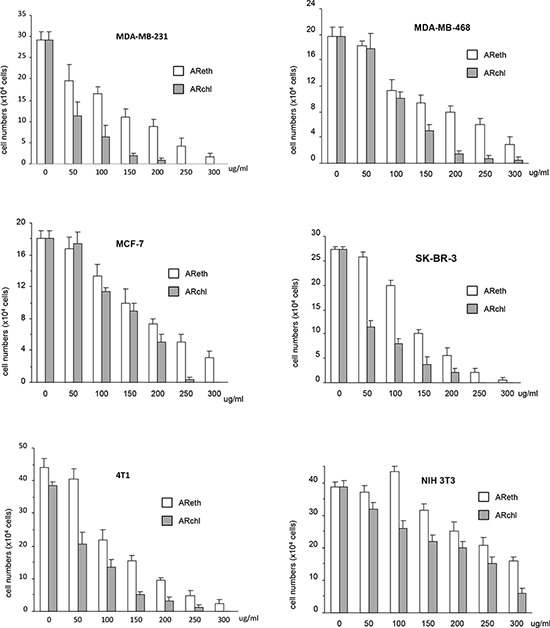
Anti-cancer activity of ethanol extract and chloroform fraction Cancer cell cultures (MDA-MB-231, SK-BR-3, MDA-MB-468, MCF-7, and 4T1) and none cancer fibroblasts NIH3T3 were treated with ethanol extract (AReth) and chloroform fraction (ARchl) for 48 hours. Cell viability was analyzed. Data represent the mean ± SD of three independent experiments.

We tested the effects of the ethanol extract (AReth) and the chloroform fraction (ARchl) on human breast carcinoma cell lines MDA-MB-231, MDA-MB-468, SK-BR-3, and MCF-7, and mouse breast cancer cell line 4T1. A normal mouse embryo fibroblast cell line NIH3T3 was used as a control. The ethanol extract (AReth) and the chloroform fraction (ARchl) were found to possess a potent effect on inducing cancer cell death (Fig. 2). As shown in the figure, AReth induced death of MDA-MB-231 in a concentration-dependent manner. Similar inhibitory effects were observed in MDA-MB-468, SK-BR-3, MCF-7, and 4T1 cells. The chloroform fraction (ARchl), which was purified from ethanol extract, displayed significantly stronger inhibitory effects on the five breast cancer cell lines than AReth at the same concentrations (Fig. 2).

The fraction was then used to purify further with the silica gel column chromatography. It was dissolved in 100 ml petroleum ether and mixed with 40 g dry silica gel (160–200 mesh). The mixture was evaporated to remove petroleum ether at 60°C. Meanwhile, silica gel (500 g) was packed into a glass column (15 × 100 cm, DxH). The evaporated ARchl-gel mixture was applied onto the top of the bed of the silica gel column, followed by elution with different mixture of eluent. The solvent system was either the mixture of petroleum ether and acetone (P/A) at the ratios of 96:4, 94:6, 93:7, 9:1, 8:2, 7:3, and 5:5, or 100% methanol, 250 ml each. A total of 891 fractions were collected. Each collection was evaporated by a rotary evaporator (Type RE3000, Shanghai Yarong Biochemistry Instrument Factory).

Each collection was analyzed by Thin Layer Chromatography (TLC). The collections showing similar bands were combined. A total of 13 combinations were reached. The anti-cancer cell activity of these combinations was tested using human breast cancer cells MT-1 and human astrocytoma cell line U87. Six combinations displayed anti-cancer activity, including TL61–69, TL110–143, TL416–451, TL452–521, TL526–535, TL536–549, in which TL61–69 showed the highest activity. The combination TL61–69 was further purified by reversed-phase high performance liquid chromatograph (RP-HPLC) (Agilent HPLC1200, USA). The column was eluted by methanol-water (at the ratio of 95:5), at the speed 4 ml/min. A fraction ARHPLC-1 was obtained, which was a white crystal with needle-like shape.

### Structural analysis of compound ARHPLC-1

**Figure 3:**
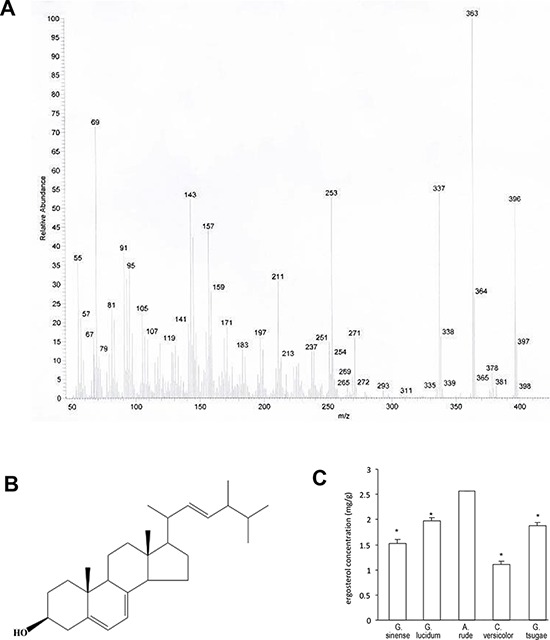
Identification of ergosterol **A.** The structure and composition of ergosterol analyzed by EI-MS. **B.** Structure of ergosterol based on the NMR data. **C.** Measurement of ergosterol concentrations from *Ganoderma sinense, Ganoderma lucidum, Amauroderma rude, Coriolus versicolor*, and *Ganoderma tsugae*.

After a series of separation and purification procedures of the silica gel column chromatography and HPLC, compound ARHPLC-1 was obtained. The structure and composition of this compound was analyzed by EI-MS and ^13^C and ^1^H NMR. The molecular formula of ARHPLC-1 was determined as C_28_H_44_O by analysis of its EI-MS (*m/z* 396(M+) (Fig. 3A) and ^13^C and ^1^H NMR data (Table 1). These data were consistent with previously reported data on ergosta-5, 7, 22-trien-3β-ol (ergosterol) [29]. Thus, ARHPLC-1 was identified as ergosta-5, 7, 22-trien-3β-ol (ergosterol), and its structure is shown in - Fig. 3B.

**Table 1:** NMR spectroscopic data of ergosterol (ARHPLC-1) in CDCL_3_

Position	ARHPLC-1 (in CDCL_3_)	
	1H (400Hz)	13C (100Hz)
1		38.3
2		32.0
3	3.63 (m,1H)	70.4
4		40.8
5		139.7
6	5.57 (d, J = 6.2Hz, 1H)	119.5
7	5.38 (t, J = 8.5Hz, 1H)	116.3
8		141.3
9		46.2
10		37.0
11		21.1
12		39.1
13		42.8
14		54.5
15		23.0
16		28.2
17		55.7
18	0.63 (s, 3H)	12.0
19	0.94 (s, 3H)	16.2
20		40.3
21	1.03 (d, J = 6.5Hz, 3H)	19.6
22	5.16 (m, 1H)	132.0
23	5.24 (m, 1H)	135.5
24		42.8
25		33.1
26	0.83 (s, 3H)	21.1
27	0.82 (d, 3H)	19.9
28	0.91(d, J = 6.9Hz, 3H)	17.5

In addition, we quantified the contents of ergosterol in various medicinal fungi, including *Ganoderma sinense, Ganoderma lucidum, Amauroderma rude, Coriolus versicolor, Ganoderma tsugae* by HPLC. The ergosterol content of *Amauroderma rude* was the highest among these popular medicinal mushrooms with a concentration of 2.58 mg/g (Fig. 3C).

### Ergosterol inhibited cancer cell migration, invasion, colony formation, and induced cancer cell apoptosis

**Figure 4:**
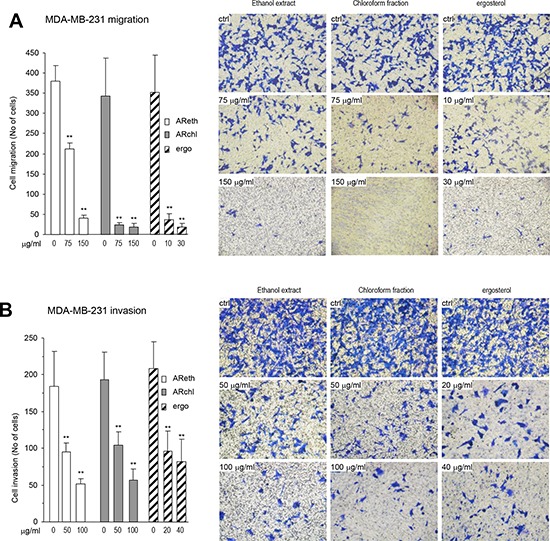
The purified ergosterol inhibited cancer cell migration and invasion **A.** Left, In Boyden chamber migration assay, the purified ergosterol was found to inhibit MDA-MB-231 cell migration significantly at very low concentrations as compared with the ethanol extract and the chloroform fraction. Right, Typical photos of cell migration through the chamber. **B.** Left, In Boyden chamber invasion assay, the purified ergosterol was found to inhibit MDA-MB-231 cell invasion significantly at very low concentrations as compared with the ethanol extract and the chloroform fraction. Right, Typical photos of cell invasion through Matrigel.

Ergosterol, the pro-vitamin D_2_, is a secondary metabolite of medicinal fungi, and shows a variety of biological activities including anti-inflammatory and anti-cancer effects [30]. We compared the effects of AReth, ARchl, and the purified ergosterol on cancer cell migration. In the Boyden chamber migration assay, we found that at the concentration of 75 μg/ml, both AReth and ARchl displayed an inhibitory effect on MDA-MB-231 cell migration, whereas the purified ergosterol exerted a significant inhibitory effect on cancer cell migration at the concentration of 10 μg/ml (Fig. 4A). In Matrigel invasion assay, we found that AReth and ARchl displayed inhibitory effect on MDA-MB-231 cell invasion at the concentration 50 μg/ml, whereas ergosterol exerted a significant inhibitory effect on cancer cell invasion at the concentration of 20 μg/ml (Fig. 4B).

**Figure 5:**
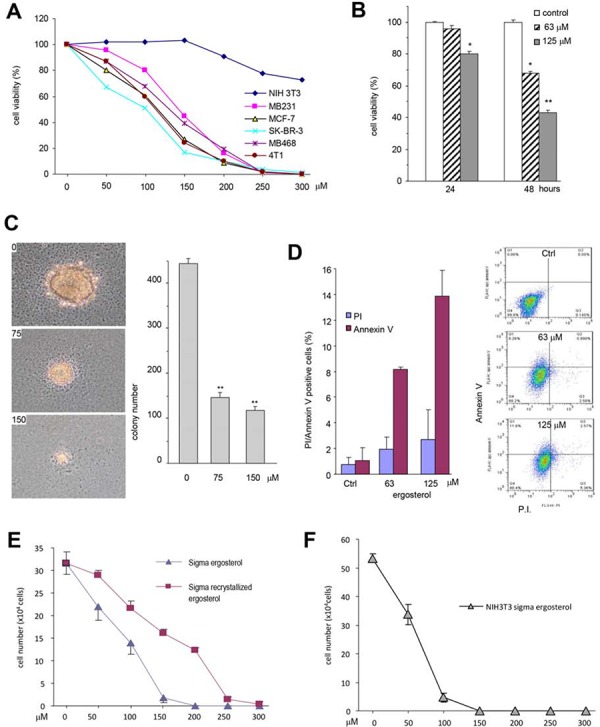
The purified ergosterol inhibited colony formation and induced cell apoptosis **A.** Cancer cell cultures (MDA-MB-231, SK-BR-3, MDA-MB-468, MCF-7, and 4T1) and none cancer fibroblasts NIH3T3 were treated with ergosterol (0–300 μM) for 48 hours. Cell viability was determined. Each experiment was repeated three times. Data represent the mean ± SD of three independent experiments. **B.** MDA-MB-231 cells were treated with ergosterol (63–125 μM) for 48 h followed by cell viability determination. Data represent the mean ± SD of three independent experiments. **C.** Colony formation in MDA-MB-231 cells was assayed with the purified ergosterol for 20 days. Left, Photos of colony sizes. Right, Number of colonies. Data represent the mean ± SD of three experiments. **D.** MDA-MB-231 cells treated with ergosterol for 12 hours were subject to cell apoptotic analysis by flow cytometry. Left, The numbers of apoptotic and necrotic cells was calculated. Apoptosis: Annexin V-positive and Prodium Iodide (PI)-negative; necrosis: PI-positive. Right, Results from one representative experiment of the cells treated with 0 (upper), 63 (middle), and 125 (lower) μM of ergosterol. **E.** MDA-MB-231 cells were treated with ergosterol (Sigma) or the product that was subject to re-crytal for brief purification for 48 h followed by cell viability analysis. Data represent the mean ± SD of three independent experiments. **F.** Cyto-toxicity of the purchased ergosterol in NIH3T3 fibroblasts.

We further tested that role of the purified ergosterol in inducing cancer cell death in a number of cancer cell lines and found that survival rates of all cancer cell lines, including MDA-MB-231, MDA-MB-468, SK-BR-3, MCF-7, and 4T1, were decreased when they were treated with 50–100 μM (20–40 μg/ml) ergosterol (Fig. 5A). However, at these concentrations, the purified ergosterol had little effect on NIH3T3 fibroblasts. In fact, the purified ergosterol displayed a significant effect on MDA-MB-231 cell survival at the concentration of 125 μM when the cells were treated for 24 hours, and at the concentration of 63 μM when the cells were treated for 48 hours (Fig. 5B).

We further tested the effect of the purified ergosterol on colony formation of MDA-MB-231 cells. The experiment showed that at the concentration of 75 μM, ergosterol inhibited colony formation in both the sizes and number of the colonies (Fig. 5C). We determined whether the ergosterol-induced cancer cell death occurred through apoptosis. At the concentration of 63 μM, ergosterol induced cancer cell apoptosis significantly (Fig. 5D). Some necrosis was also detected but not significantly.

Since synthetic ergosterol is available commercially, we tested the effect of the commercially available ergosterol on cancer cell survival. It was found that the synthetic ergosterol induced cell death more potent than the purified ergosterol (Fig. 5E). To test if there was some impurity in the product which was of cytotoxicity, we tested the effect of the product on the non-cancer cell line NIH3T3 fibroblasts. We found that extensive cell death was detected when the cells were treated with 50 μM of synthetic ergosterol (Fig. 5F). We briefly purified the ergosterol by recrystallization using ethanol precipitation method. The purified synthetic ergosterol was then tested on MDA-MB-231 cells and had a similar effect on MDA-MB-231 cells as the naturally purified ergosterol (Fig. 5E).

### Ergosterol treatment prolonged animal survival

**Figure 6:**
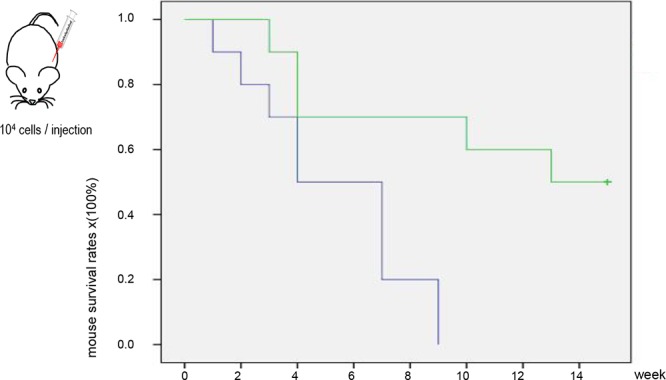
Ergosterol treatment prolonged animal survival Kaplan-Meier of survival curves for mice injected intraperitoneally with B16 cells comparing the ergosterol-treated group with control, non-treated group (log-rank test, *p* < 0.01).

Since the amount of ergosterol purified from *Amauroderma rude* was not sufficient to conduct *in vivo* experiment, we used the recystallized ergosterol for the *in vivo* assay to inhibit tumor growth. While we used human and mouse cancer cell lines to test the anti-cancer effect of ergosterol on different cancer cell lines, we only used mouse cancer cells B16 to perform the *in vivo* experiments. In this way, regular mice (Strain Balb/c) with normal immune system could be used, since ergosterol may be of potentiation ability for the immune system. We found that mice treated with ergosterol displayed a significantly longer survival time than the control mice according to the Kaplan-Meier analysis of survival curves (Fig. 6). The mean survival time of control groups was 5.3 weeks (range 1–9 weeks), while the mean survival time of the treated groups was 10.9 weeks (range 3–15 weeks). After 15 weeks, five mice from the ergosterol-treated group were still alive in good health conditions, while the experiment was terminated. This data suggest that ergosterol had anti-cancer effects *in vivo* leading to a longer lifespan.

### Ergosterol treatment increased Foxo3 expression in breast cancer cells

We examined whether ergosterol induced cancer cell apoptosis by up-regulating expression of tumor suppressors. The forkhead box O3, also known as Foxo3, is a transcription factor encoded by the *FOXO3* gene [31]. Foxo3 plays pivotal roles in tumorigenesis and drug resistance [32–34]. Foxo3 deregulation is essential in the development of cancer [35].

**Figure 7:**
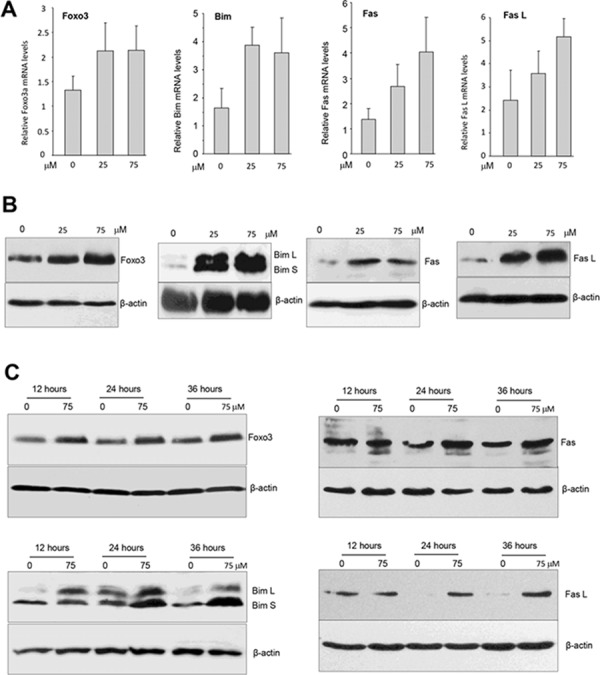
Ergosterol up-regulated multiple tumor suppressors **A.** Total RNAs isolated from MDA-MB-231 cells treated with ergosterol for 12 hours were subject to real-time PCR to analyze expression of tumor suppressors of Foxo3, Bim, Fas, and Fas L. **B.** Cell lysates prepared from MDA-MB-231 cells treated with ergosterol for 20 hours were subject to Western blot analysis for expression of Foxo3, Bim, Fas, and Fas L. Ergosterol promoted expression of these tumor suppressors. **C.** The cells were also treated with ergosterol for different time points and analyzed for expression of Foxo3, Bim, Fas, and Fas L.

We examined the effect of ergosterol on Foxo3 expression by real-time PCR using total RNAs isolated from MDA-MB-231 cells treated with or without ergosterol using the primers listed (Table 2). We found that Foxo3 mRNA expression was significantly up-regulated as compared with the control (Fig. 7A). Western bolt analysis revealed that Foxo3 protein also increased in the cells treated with ergosterol (Fig. 7B).

**Table 2:** Sequences of primers used in the study

Gene	Primer sequence
*Bim*	Forward 5′ - TGC CAG GCC TTC AAC CAC TAT CTC -3′
	Reverse 5′ - AGA GGG AGA GAG GTG GCT GTG GCT -3′
*Fas*	Forward 5′ - TGA ACA TGG AAT CCA AGG AA -3′
	Reverse 5′ - ATA GTG GAT ATT TAC TCA AG -3′
*FasL*	Forward 5′ - GCC TGG TCA AAG GAG GCC ACC ACC -3′
	Reverse 5′ - GTA GGT GGA AGA GCT GAA ACA TCC -3′
*Foxo3a*	Forward 5′ - GCA AGA GCT CTT GGT GGA TCA TCA A -3′
	Reverse 5′ - TGG GGC TGC CAG GCC ACT TGG AGA G -3′
*U6*	Forward 5′ - CAC CGT GCT TTC GGC AGC ACA TAT AC -3′
	Reverse 5′ - ACC GTG CAC CGG CGT ATA AAC GTG GTG TA -3′

Foxo3 has been shown to activate pro-apoptotic genes such as *FasL, Bad, Bim, Trail* [36–38]. Bim is a Foxo3 target gene and is one of the BH3-only protein, and its upregulation induces cell apoptosis [36, 37]. There are three major isoforms of Bim including BimEL, BimL, and BimS as a result of alternative splicing [39]. We found that ergosterol treatment increased the levels of Bim L and Bim S in a concentration-dependent manner (Fig. 7B).

Fas L (Fas ligand) is a ligand of a death receptor on the cell surface, linking with its receptor Fas triggers the cell extrinsic apoptotic pathway. It has been reported that Foxo3 increased expression of Fas L and concurrent expression of Fas to induce cell apoptosis in response to environmental stressor [40]. We measured mRNA levels of Fas and Fas L in the cells treated with or without ergosterol and found that the treated cells showed increased expression of the mRNA levels of Fas and Fas L (Fig. 7A). Consistent with this result, we detected increased expression of the protein levels of Fas and Fas L by Western blotting probing with antibodies against Fas and Fas L. Furthermore, we also treated the cells with ergosterol for different time periods and confirmed up-regulation of Foxo3, Bim L, Bim S, Fas, and Fas L (Fig. 7C). Our results showed that treatment with ergosterol up-regulated multiple tumor suppressors.

## DISCUSSION

We previously compared the anti-cancer effect of 13 different medicinal mushrooms and reported that the water extract of *Amauroderma rude* possesses the most effective capacity in inducing cancer cell death [16]. It has been thought that mushroom polysaccharides are the major component in the anti-cancer activities through activation of the immune response of the host organism. We quantitated the ploysaccharides contents of the 13 medicinal mushrooms and found that the amount of ploysaccharides did not match the anti-cancer effect of the mushrooms [16]. We reasoned that some small molecules might play key role in inducing cancer cell death.

In this study, we extracted small molecules from *Amauroderma rude* with ethanol. After a series of fractionation, chromatography, and HPLC purification, we identified ergosterol as the anti-cancer molecule. In the purification process, the sensitivity of the system to screen for anti-cancer ingredients is critical. The important factors for effective identification of the compounds are the time lines and the amounts of anti-cancer ingredients used. The assay should be sensitive with only a small amount of the ingredients and the results can be obtained within 1–2 days. In the initial screening, we found that the ethanol extract exerted a strong effect on inducing cancer cell death. This result suggests that small molecules, which were extracted by ethanol, indeed possessed potent effects on inducing cancer cell death. With a number of cancer cell lines to test the effects of the anti-cancer compounds, which were developed by us previously [16], we were able to identify ergosterol as the major molecule in inducing cancer cell death.

It should be mentioned that there were other ingredients and molecules that possessed anti-cancer activity. For example, when we fractionated the ethanol extract, we could only use the fraction with the highest anti-cancer activity for further purification. This relied on the total anti-cancer activity of each fraction, rather than the anti-cancer activity of a single molecule. It is possible that one fraction might contain some molecules with very high anti-cancer activity, but still not be selected because this fraction contained fewer such molecules of anticancer activity. When we combined the 891 fractions into 13 combinations based on their migration patterns in Thin Layer Chromatography, we could only use two cell lines, MT1 and U87, which have been found to be highly sensitive to anti-cancer ingredients [16] to save samples. There could be other cancer cell lines that are more sensitive to some other ingredients. In the 13 combinations, we found at least 6 of them possessed anti-cancer activity, but we could only identify the one with the highest activity. Other combinations could contain novel molecules with potent anti-cancer activity. This awaits further investigation.

Ergosterol had long been recognized as an important bioactive compound isolated from other sources including medical fungi [41–45]. Yazawa and co-workers showed that ergosterol, isolated from *Polyporus*, provides significant protection against the promotion of bladder tumor on rats which have been induced by many types of promoters in the environment [46]. Takaku et al. reported that ergosterol isolated from *Agaricus blazei* directly inhibited angiogenesis induced by solid tumors, using neovascularization model induced by Lewis lung carcinoma cell-packed chambers and Matrigel [47]. Zhang et al. reported that ergosterol isolated from *Agrocybe aegerita* showed inhibitory activity towards the COX-II enzyme to reduce inflammation and promote cancer prevention [48]. In our study, we adapted a mouse model to investigate the effect of ergosterol on the survival of mice bearing aggressive cancer cells B16 which causes animal death. Our results showed that ergosterol prolonged survival rates of tumor-bearing animals. This may be of clinical significance.

Furthermore, we explored the molecular mechanism underlying this effect. Our data showed that Foxo3 was up-regulated after ergosterol treatment in breast cancer cells (MDA-MB-231) when the cells underwent apoptosis. It has been identified that the over-expression of Foxo3 inhibited tumor growth *in vitro* and tumor development *in vivo* in many cancer [49, 50]. Therapy-resistant acute lymphoblastic leukemia (ALL) cells were shown to inactivate Foxo3 activity to escape apoptosis induction [51]. Foxo3 can induce cell apoptosis by directly activating the extrinsic apoptotic pathway through up-regulation of death receptor ligand Fas L and the intrinsic apoptotic pathway with up-regulation of pro-apoptotic BH-3 only Bim [52]. Our results showed that Bim and Fas L were indeed up-regulated after ergosterol treatment at the transcription and translation levels. These data provided evidence that Foxo3 directly activates its target genes Bim and Fas L to triggered cancer cell apoptosis via the mitochondria-dependent intrinsic pathway and the death receptor ligand-dependent extrinsic pathway, after ergosterol treatment.

In summary, we investigated the anti-cancer activity of *Amauroderma rude*. Small molecules of anti-cancer activity were obtained in the ethanol extract, which were then detected in the chloroform fraction. Ergosterol, which is the major active component, was purified by gel silica, chromatography, HPLC purification. We further showed that ergosterol prolonged animal survival by up-regulating Foxo3 and Foxo3 downstream molecules Bim, Fas, and Fas L. These results provide new insight for future development of *Amauroderma rude* as an effective and safe medical mushroom for use by cancer patients. It also provides evidence allowing us to further understand the molecular mechanism by which ergosterol exerts its anti-cancer activity. Future work could lead to synthesis and modification of ergosterol structure to improve its effective therapy in clinical settings.

## MATERIALS AND METHODS

### Extraction, isolation and purification of anti-cancer compounds

The dry fruit bodies of *Amauroderma rude* (10 kg) were pulverized to powder. The powder was soaked in 95% ethanol at a ratio of 1:15 (w/v), and extracted by refluxing three times at 80°C, 2 hours each. The extracted solution was filtered and concentrated under a vacuum to remove the solvents. A yield of 520 g was obtained.

The ethanol extract was subject to fractionation and purification using different polarity solvent and silica gel column chromatography. In brief, the ethanol fraction was dispersed in distilled water. The resulting suspension was fractionated by petroleum ether, chloroform, ethyl acetate, and water-saturated butanol in turn. Each fraction was collected and evaporated. The anti-tumor activity of all fractions was tested with human breast cancer cell line MT1 and human astrocytoma cells U87 cells. The bioactive components were subject to further purification. This included silica gel chromatography, Thin Layer Chromatography (TLC), and reversed-phase high performance liquid chromatograph (RP-HPLC). The detailed steps are provided in the results section.

### MS and NMR analysis

For MS identification, the compound ARHPLC-1 was dissolved in methanol and recorded on EI-mass spectrometer (DSQ II, Thermo, USA). For NMR measurements, ARHPLC-1 was dissolved with CDCL3. The NMR spectra were recorded on BRUKER AVANCE IIIT 400HD (Bruker BioSpin, Switzerland).

### Cell migration

The Boyden transwell inserts were placed onto each well of the 24-well tissue culture plates, to which 500 μl medium containing 10% fetal bovine serum (FBS) had been added. MDA-MB-231 cells (1 × 10^4^ cells in 100 μl serum-free medium containing different concentrations of AReth, ARchl, or ergosterol) were loaded into each transwell, followed by incubation at 37°C for 6 hours. The cells in the transwell inserts were fixed with cool methanol for 15 min and stained with Coomassie Brilliant Blue. Cells that stayed on the upper surface of the inserts were removed. Cells that migrated through the transwells and spread onto the lower surface of the transwells were photographed under a light microscope and counted from representative areas for quantification.

### Cell invasion

The invasion assay was performed as described [53]. In brief, the transwell inserts were loaded with 80 μl of Matrigel (1:8 dilution) and incubated at 37°C for 30 min allowing a gel-bed to form. The inserts were then placed onto the wells of a 24-well plate, to which 500 μl medium supplemented with 10% FBS had been added. MDA-MB-231 cells (1 × 10^4^ cells in 100 μl serum-free medium containing different concentrations of AReth, ARchl, or ergosterol) were gently loaded on top of each gel bed, followed by incubation at 37°C for 36 hours. The transwell inserts were fixed with cool methanol for 15 min and stained with Coomassie Brilliant Blue. The upper Matrigel layer and cells were removed. Cells that invaded through the Matrigel and spread onto the lower surface of the inserts were photographed under a light microscope and counted from representative fields for quantification.

### Viability and cell death assay

Human breast carcinoma cell lines (MB231, MB468, SK-BR-3, and MCF-7), and mouse breast cancer cell line 4T1 were used to test the effects of each sample in inducing tumor cell death. In brief, cells (1 × 10^5^ cells/ml) were seeded in 24-well tissue culture plates in DMEM/RPMI1640 supplemented with 10% FBS, 100U/ml penicillin/streptomycin at 37°C, 5% CO_2_. Four hours after cell inoculation, the ethanol extract (AReth), chloroform fraction (ARchl) and a pure compound (ARHPLC-1 or ergosterol) were added to the cultures at different concentrations and incubated for 48 hours. Cell viability was analyzed by trypan blue staining.

### Colony growth assay

Colony growth assay was performed using previously described method [54, 55]. Briefly, MDA-MB-231 cells (500 cells/well) were seeded in 6-well plates and cultured in 0.25% low melting agarose gel containing ergosterol. Two weeks after cell growth, colonies were counted, fixed, stained, and photographed under a microscope.

### Flow cytometry

Cell apoptosis was analyzed by Annexin V apoptosis detection kit APC (eBioscience, Inc., USA) as described [56, 57]. In brief, MDA-MB-231 cells were treated with ARHPLC-1 (ergosterol) at different concentrations. Six hours after cell incubation, the cells were washed, trypsinized, collected, and resuspended in 100 μl binding buffer, followed by incubation with Annexin V (APC) in the dark at room temperature for 15 minutes. The cells were then washed, resuspended in 400 μl binding buffer, to which 5 μl of Propidium Iodide staining solution were added. The cells were analyzed by flow cytometry (FACS Calibur, Becton, Dickinson and Company. USA). The cells showing Annexin V-positive and PI-negative were counted as apoptotic, whereas PI-positive staining was counted as necrotic cells.

### Western blotting

After being treated with the purified compound of *Amauroderma rude*, cells were lysed by the lysis buffer containing protease inhibitor (CALBIOCHEM, USA) on ice for 30 min and centrifuged to obtain clear cell lysates. Protein concentrations were determined by Bio-Rad Dc protein assay. Samples with equal contents of proteins were separated using SDS-PAGE and transferred onto NC membranes (Perkin Elmer, USA). After blocking with 5% milk, the membranes were incubated with primary antibodies at 4°C overnight. Next day, the membranes were washed three times, incubated with secondary antibodies for 2 hours, and washed three times again. The membranes were developed with the ECL kit (Millipore, USA) and visualized using the Kodak Image Station 4000R.

### Real-time PCR

Total RNA was extracted using Total RNA Mini kit (Frogga Bio Inc, Canada) as described [58]. RNA concentrations were measured by NanoDrop 2000c UV-VIS Spectrophotometer (Thermo Scientific, USA) under 260 nm. Equal amount of RNA was reverse-transcribed using SuperScript Ш First-Strand Synthesis System (Invitrogen, USA) to obtain cDNA. The cDNA was diluted (1:5 dilutions) and used as templates for real-time PCR using a SYBR Green PCR Kit (QIAGEN). The PCR was performed for 40 cycles: denaturation at 95°C for 5 sec, annealing at 59°C for 30 sec, and elongation at 72°C for 30 sec (ABI PRISM 7700 Sequence Detection System, USA). The mRNA levels of *Foxo3a, Bim, Fas* and *Fas L* were analyzed using *U6* as an internal control. All tests were performed in triplicate.

### Animal survival assay

Four-week old mice were obtained from Charles River. Animals were kept in the Animal Core Facility of Sunnybrook Research Institute for one week before experimentation. The mice were randomly divided into two groups. Twenty mice (approximately 20 g each) were injected intraperitoneally with B16 cells (1 × 10^4^ cells/mouse). Next day after cell implantation, ARHPLC-1 (ergosterol) was injected intraperitoneally at a dose of 50 mg/kg/mouse. This was repeated every other day for up to 3 months. The control group was mice injected with 0.9% sodium chloride at the same volume on the same schedule. All animal experiments were conducted according to the guideline approved by the Animal Care Committee at Sunnybrook Research Institute.

### Statistical analysis

The results of all the experiments were subject to statistical analysis by *t*-test. The level of significance was set at *p* < 0.05 and *p* < 0.01 respectively. Animal survival was analyzed using the Kaplan Meier survival curves with log-rank analysis.
